# Rapid Fatal Outcome from Pulmonary Arteries Compression in Transitional Cell Carcinoma

**DOI:** 10.1155/2009/579407

**Published:** 2010-01-06

**Authors:** Ioannis A. Voutsadakis, George Masouris, Konstantinos Tsapakidis, Christos N. Papandreou

**Affiliations:** Department of Medical Oncology, University Hospital of Larissa, Larissa 41110, Greece

## Abstract

Transitional cell carcinoma of the urinary bladder is a malignancy that metastasizes frequently to lymph nodes including the mediastinal lymph nodes. This occurrence may produce symptoms due to compression of adjacent structures such as the superior vena cava syndrome or dysphagia from esophageal compression. We report the case of a 59-year-old man with metastatic transitional cell carcinoma for whom mediastinal lymphadenopathy led to pulmonary artery compression and a rapidly fatal outcome. This rare occurrence has to be distinguished from pulmonary embolism, a much more frequent event in cancer patients, in order that proper and prompt treatment be initiated.

## 1. Introduction

Cancer is a prothrombotic condition that predisposes patients to various thromboses such as lower extremities thromboses and pulmonary embolism [1, 2]. Obstruction of blood flow from external compression, frequently from lymph node masses, is also common, especially malignancy of the lower abdomen and pelvis which can cause lower extremity edema and in the mediastinum which can cause superior vena cava syndrome from mediastinal lymphadenopathy [3]. In contrast, compression of pulmonary vessels from mediastinal lymphadenopathy is less common but is important to correctly diagnose because the treatment and prognosis are different from those of pulmonary embolism. We report the case of a man with metastatic bladder carcinoma who had massive mediastinal lymphadenopathy causing external pulmonary artery compression leading to a rapidly fatal outcome.

## 2. Case Report

A 59-year-old man was admitted to the hospital with severe fatigue and dyspnea at rest. He had undergone a radical cystectomy with neobladder construction for transitional cell carcinoma of the urinary bladder seven months previously and had received five cycles of adjuvant dose-dense MVAC (Methotrexate, Vinblastine, Adriamycin, and Cisplatin) chemotherapy after the operation. A few days before admission, restaging CTs showed multiple liver metastases, two right kidney lesions, a lytic lesion in the thoracic spine, massive right hilar lymphadenopathy, right pelvic lymphadenopathy, and a right pleural effusion.

**Figure 1:**
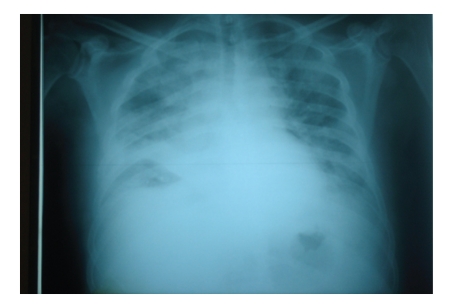
Chest X-ray showing a right middle lobe atelectasis, bilateral infiltrates, and mediastinal lymphadenopathy.

**Figure 2:**
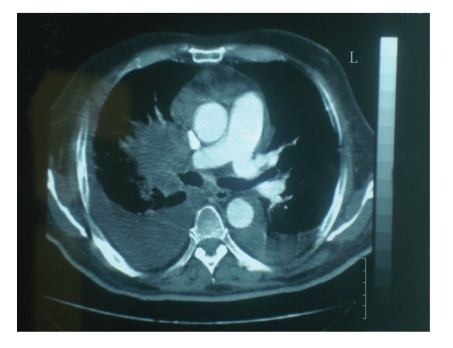
CT arteriography at the carina showing occlusion of the right pulmonary artery due to external compression from a mass of 6.8 cm and atelectasis. No intraarterial thrombus is seen. Left pulmonary artery is nearly completely occluded from lymph node mass of 2.3 cm. Extensive mediastinal lymphadenopathy was also seen. Pleural effusions are also evident.

On admission, the patient was pale, with dyspnea at rest, tachypnea, and tachycardia, but he had no chest pain, cough or hemoptysis. His blood pressure was 110/70 mmHg and the O_2_ saturation was 97% when the patient was on room air. The pO_2_ was 83 mmHg and pCO_2_ was 30 mmHg. No deep vein thrombosis was clinically evident in the lower extremities. Laboratory evaluation showed a leukocytosis at 45,000 /*μ*L with neutrophilia at 43,700 /*μ*L and lymphopenia at 500 /*μ*L as well as a hemoglobin of 9.5 gm/dL and platelets at 66,000 /*μ*L. Other abnormal values from the biochemical evaluation included the alkaline phosphatase at 218 IU/L (normal range 35–104 IU/L), the LDH at 590 IU/L (normal range 135–225 IU/L), CRP at 12.66 mg/dL (normal range 0–10 mg/dL), INR at 1.52, and fibrinogen at 567 mg/dL. A chest X-ray showed right middle lobe atelectasis and bilateral infiltrates (Figure 1). ECG displayed no signs of right ventricular strain. CT angiography of pulmonary arteries showed absence of arterial thrombi in the vessels but confirmed extensive mediastinal and hilar lymphadenopathy obstructing and impeding blood flow in both pulmonary vessels (Figure 2). In preparation for emergency radiation treatment, the patient had a cardiopulmonary arrest and could not be resuscitated.

## 3. Discussion

The constellation of symptoms as described in this patient's acute presentation is suggestive of acute pulmonary embolism, a condition not uncommon in patients with neoplastic diseases [4]. Cancer is a prothrombotic state and cancer patients frequently have other causes promoting thromboses such as reduced mobility, central catheters, and surgery [1]. In addition to pulmonary embolism due to thrombosis, cancer patients may suffer from embolism due to neoplastic emboli, a condition termed pulmonary tumor embolism. This has been seen in up to 26% of cancer patients in autopsy studies [5, 6] and has been documented in transitional cell carcinoma of the bladder [7].

 Contrast-enhanced computer tomographic (CT) arteriography of the pulmonary vessels and ventilation-perfusion scintigraphy (V/Q scanning) are the studies used for diagnosis of pulmonary embolism [8]. Contrast-enhanced CT arteriography has the advantage over ventilation-perfusion scintigraphy in being able to visualize and characterize nonvascular structures. It is also particularly useful in the presence of pleural effusions, a common occurrence in malignancy, where V/Q scanning loses its diagnostic accuracy. Signs of pulmonary embolism with CT arteriography include intraluminal filling defects, total cutoff of vascular enhancement, enlargement of the occluded vessel, pleural-based wedge-shaped areas with no contrast enhancement, and linear atelectasis [8, 9]. In contrast, CT arteriography cannot distinguish pulmonary embolism from the rare intra-arterial tumor masses [10]. In this case diagnosis may require intravascular sampling or even a minithoracotomy [10]. CT arteriography in our patient did not show pulmonary embolism but instead revealed external pressure on the pulmonary arteries from metastatic adenopathy, illustrating this advantage of CT arteriography.

 Lymphadenopathy compressing the circulation of lower extremities is a common problem in patients with pelvic malignancies while mediastinal lymphadenopathy, most commonly from lung cancers, is a common cause of the superior vena cava syndrome [3]. Bladder cancer has a tendency to metastasize to intraabdominal lymph nodes, liver, bones, and lung [11–13] while thoracic lymphadenopathy is also common although less than intraabdominal lymphadenopathy. Mediastinal metastases from transitional cell carcinoma of the bladder either are asymptomatic or may more commonly produce the superior vena cava syndrome or dysphagia from esophageal invasion or compression [14]. Pulmonary arterial compression as seen in our patient is far less common and indeed this is to the best of our knowledge the first case with this occurrence reported in the English literature. Nevertheless it needs to be included in the differential diagnosis of a cancer patient presenting with symptoms suggestive of acute pulmonary embolism because the treatments of the two conditions are different. Prompt initiation of anticoagulation is mandatory in a patient diagnosed with pulmonary embolism while treatment for external compression includes radiation therapy with the possible addition of steroids to reduce concomitant soft tissue edema and improve reactive bronchoconstriction. As the molecular biology of metastatic bladder cancer becomes clearer [15] and therapeutic options increase with both conventional chemotherapy and targeted therapies [16–18], successful management of these patients may become more relevant.
